# Critical review: vegetables and fruit in the prevention of chronic diseases

**DOI:** 10.1007/s00394-012-0380-y

**Published:** 2012-06-09

**Authors:** Heiner Boeing, Angela Bechthold, Achim Bub, Sabine Ellinger, Dirk Haller, Anja Kroke, Eva Leschik-Bonnet, Manfred J. Müller, Helmut Oberritter, Matthias Schulze, Peter Stehle, Bernhard Watzl

**Affiliations:** 1 Department of Epidemiology, German Institute of Human Nutrition, Potsdam-Rehbrücke, Germany; 2 Science Department, German Nutrition Society, Bonn, Germany; 3 Department of Physiology and Biochemistry of Nutrition, Max Rubner-Institut, Karlsruhe, Germany; 4 Department of Nutrition and Food Science, University of Bonn, Bonn, Germany; 5 Nutrition and Food Research Centre, Chair for the Biofunctionality of Food, Technical University of Munich, Freising-Weihenstephan, Germany; 6 Department of Nutritional, Food and Consumer Sciences, Fulda University of Applied Sciences, Fulda, Germany; 7 Institute of Human Nutrition and Food Science, Christian-Albrechts-University Kiel, Kiel, Germany; 8 Department of Molecular Epidemiology, German Institute of Human Nutrition, Potsdam-Rehbrücke, Germany

**Keywords:** Vegetables, Fruit, Prevention, Chronic diseases, Epidemiology

## Abstract

**Background:**

Vegetables and fruit provide a significant part of human nutrition, as they are important sources of nutrients, dietary fibre, and phytochemicals. However, it is uncertain whether the risk of certain chronic diseases can be reduced by increased consumption of vegetables or fruit by the general public, and what strength of evidence has to be allocated to such an association.

**Methods:**

Therefore, a comprehensive analysis of the studies available in the literature and the respective study results has been performed and evaluated regarding obesity, type 2 diabetes mellitus, hypertension, coronary heart disease (CHD), stroke, cancer, chronic inflammatory bowel disease (IBD), rheumatoid arthritis (RA), chronic obstructive pulmonary disease (COPD), asthma, osteoporosis, eye diseases, and dementia. For judgement, the strength of evidence for a risk association, the level of evidence, and the number of studies were considered, the quality of the studies and their estimated relevance based on study design and size.

**Results:**

For hypertension, CHD, and stroke, there is convincing evidence that increasing the consumption of vegetables and fruit reduces the risk of disease. There is probable evidence that the risk of cancer in general is inversely associated with the consumption of vegetables and fruit. In addition, there is possible evidence that an increased consumption of vegetables and fruit may prevent body weight gain. As overweight is the most important risk factor for type 2 diabetes mellitus, an increased consumption of vegetables and fruit therefore might indirectly reduces the incidence of type 2 diabetes mellitus. Independent of overweight, there is probable evidence that there is no influence of increased consumption on the risk of type 2 diabetes mellitus. There is possible evidence that increasing the consumption of vegetables and fruit lowers the risk of certain eye diseases, dementia and the risk of osteoporosis. Likewise, current data on asthma, COPD, and RA indicate that an increase in vegetable and fruit consumption may contribute to the prevention of these diseases. For IBD, glaucoma, and diabetic retinopathy, there was insufficient evidence regarding an association with the consumption of vegetables and fruit.

**Conclusions:**

This critical review on the associations between the intake of vegetables and fruit and the risk of several chronic diseases shows that a high daily intake of these foods promotes health. Therefore, from a scientific point of view, national campaigns to increase vegetable and fruit consumption are justified. The promotion of vegetable and fruit consumption by nutrition and health policies is a preferable strategy to decrease the burden of several chronic diseases in Western societies.

## Introduction

Vegetables and fruit are extremely important in human nutrition as sources of nutrients and non-nutritive food constituents as well as for the reduction in disease risks. While their importance as sources of nutrients and non-nutritive food constituents is generally accepted, there are still uncertainties regarding their relevance for the prevention of diseases. For this reason, it has to be determined first, for which diseases studies have detected an association between the consumption of vegetables and fruit and the risk of disease, and subsequently, how this association has to be judged. This information provides an important basis to judge the preventive potential of a diet rich in vegetables and fruit. For example, this would allow to estimate the changes regarding the incidence of certain diseases that have to be expected if, for example, the “5 a day” recommendation on the consumption of about 650 g vegetables and fruit per day would be implemented by the majority of subjects in Germany.

Therefore, a working group within the German Nutrition Society (DGE) was established in 2006 with the aim to evaluate the evidence on the role of vegetables and fruit regarding the prevention of certain chronic diseases. The available data were recorded by comprehensive literature search, and the respective strength of the evidence was determined by criteria defined in advance. This evaluation of the evidence was published in 2007 in German as a DGE-statement [1].

As further studies on the association between the consumption of vegetables and fruit and the risk of disease have been published since 2007, it was necessary to update the statement. Therefore, the available data on the diseases selected in 2007 once again were comprehensively recorded with focus on prospective epidemiologic observational and intervention studies, and based upon these study data, the evidence regarding a preventive effect was judged.

## Methods

The review is based upon the comprehensive analysis of the epidemiological studies available in the literature on vegetables and fruit. The authors agreed at the beginning of the study to cover the same list of diseases that were included into the DGE-statement from 2007 [1] since no other disease group than the previously selected appeared to be newly associated with consumption of vegetables and fruit. For each disease under consideration, a literature search in the NCBI PubMed database was done that included the literature until December 2010. The search strategy comprised the keywords “fruit” and “vegetables” and the various disease outcomes (Table 1). The type of studies that are searched for differed according to endpoint. Based on the experience from the DGE-statement from 2007 [1], for some endpoints such as type 2 diabetes, hypertension, coronary heart disease, stroke, and cancer, the search had been restricted to intervention and cohort studies. For other endpoints, all types of epidemiological studies had been looked at in the database. In addition to the studies identified in the newly conducted literature search, the references in relevant publications were reviewed in order to have identified all of the studies. Also, the literature research in conjunction with the DGE-statement from 2007 [1] was taken into account. Furthermore, studies that have been published until April 2011 were included in the review if they contain new information relevant for the judgement of the evidence.

**Table 1:** NCBI search terms and disease endpoints of the review

NCBI search terms for the exposure (humans only)	Selected disease end points	NCBI search terms for the disease endpoints
*Fruit* *OR vegetable*		
Intervention OR cohort studies	Obesity	Weight change OR weight gain OR obesity
Meta-analysis OR review		
Intervention OR cohort studies	Type 2 diabetes mellitus	Diabetes OR insulin sensitivity OR insulin resistance OR prediabetes OR impaired glucose tolerance OR impaired fasting glucose OR fructosamine OR A1c
Meta-analysis OR review		
Intervention OR cohort studies	Hypertension	Hypertension OR blood pressure
Meta-analysis OR review		
Intervention OR cohort studies	Coronary heart disease	Coronary heart disease OR CHD OR cardiovascular disease OR CVD OR coronary artery disease OR myocardial infarction
Meta-analysis OR review		
Intervention OR cohort studies	Stroke	Stroke OR cerebrovascular
Meta-analysis OR review		
Risk factor AND cohort studies	Cancer	Cancer
Meta-analysis OR review		
Intervention OR cohort OR case–control OR cross-sectional studies	Chronic inflammatory bowel diseases	Chronic inflammatory bowel diseases OR Crohn’s disease OR ulcerative colitis
Meta-analysis OR review		
Intervention OR cohort OR case–control OR cross-sectional studies	Rheumatoid arthritis	Rheumatoid arthritis
Meta-analysis OR review		
Intervention OR cohort OR case–control OR cross-sectional studies	Chronic obstructive pulmonary disease (COPD)	Chronic obstructive pulmonary disease
Meta-analysis OR review		
Intervention OR cohort OR case–control OR cross-sectional studies	Asthma	Asthma
Meta-analysis OR review		
Intervention OR cohort studies	Osteoporosis	Osteoporosis
Meta-analysis OR review		
Intervention OR cohort OR case–control OR cross-sectional studies	Eye diseases	Age-related macular degeneration OR glaucoma OR diabetic retinopathy OR cataract
Meta-analysis OR review		
Intervention OR cohort OR case–control OR cross-sectional studies	Dementia	Dementia OR Alzheimer
Meta-analysis OR review		

The scheme of generating the level of evidence associated with each study according to its study design followed the considerations of the WHO [2] and the evidence-based guidelines for the prevention of nutrition-related diseases of the DGE [3]. Intervention studies were given the highest level of evidence, followed by methodologically well-conducted cohort studies (Table 2). Meta-analyses are rated higher than individual studies among its level. In theory, for deriving the relative risk, case–control studies have similar strength than cohort studies. In practice, however, compared with cohort studies, they have the disadvantage of recall and selection bias. This especially applies to case–control studies in the field of nutrition. Therefore, they are rated with a lower level of evidence than cohort studies. Likewise, cross-sectional studies are allocated with a low level of evidence because their study design does not show a clear temporal connection between the investigated nutritional factor and the disease.

**Table 2:** Classification and judgement of the strength of the evidence

Level of evidence	Type of study/publication	Strength of the evidence
Ia	Meta-analysis of randomised, controlled intervention studies	Convincing^a^/
Ib	Randomised controlled intervention studies	Probable^b^/
Ic	Non-randomised/non-controlled intervention studies (if well-designed, otherwise level IV)	Possible^c^
		Evidence
IIa	Meta-analysis of cohort studies	Convincing^a^/
IIb	Cohort studies	Probable^b^/
		Possible^c^/
		Insufficient^d^
		Evidence
IIIa	Meta-analysis of case–control studies	Probable^b^/
IIIb	Case–control studies	Possible^c^/
		Insufficient^d^
		Evidence
IV	Non-analytic studies	Possible^c^/
	(Cross-sectional studies, case reports etc.)	Insufficient^d^
	Reports/opinions of expert committees or consensus conferences, which did not determine the strength of the evidence, and/or clinical experience of respected authorities	Evidence

^a^Is assigned if there are a considerable number of studies including prospective observational studies and, wherever possible, randomised controlled intervention studies of sufficient size, duration and quality with consistent results

^b^Is assigned if epidemiological studies show fairly consistent relations between factor and disease, but there are noticeable weaknesses regarding the evidence or there is evidence of an opposite relation, which does not allow a definite judgement

^c^Is assigned if the results on an association between exposure and target disease are mainly based upon case–control studies and cross-sectional studies. There are only insufficiently performed controlled intervention studies, observational studies, or non-controlled clinical trials

^d^Is assigned if there are a few study results that indicate an association between a factor and a disease, but they are not sufficient to establish the relation. There is only limited or no evidence from randomised intervention studies

Based on the number of available studies and their classifications of level of evidence, the judgement of the strength of the evidence was performed (Table 2). In total, four categories of the strength of the evidence were used [3], termed as convincing, probable, possible, and insufficient. Table 2 shows the connection between the levels of evidence of the studies and the overall strength of the evidence. In addition to the relation between the level of evidence of the studies and the strength of the evidence, there were also further specifications that determined the strength of the evidence:

### Convincing evidence regarding a preventive effect or a lack of an association

The strength of evidence was judged as “convincing” if at least 2 studies of highest quality (level of evidence I) showed consistent results. If the studies showed methodological weakness or were only cohort studies, the minimum number of intervention studies was raised to 5. However, for this strength of the evidence, it was required that the question has been extensively investigated and that there were a lot of results from different study populations including comprehensive data on consumption. Results from cohort studies should have been confirmed by intervention studies with intermediary markers regarding causality. Ideally, a meta-analysis of the present studies is available that did neither indicate heterogeneous study results nor include a high percentage of study results with opposite effects.

### Probable evidence regarding a preventive effect or a lack of an association

The strength of the evidence was judged as “probable” if epidemiological studies showed consistent relations between factor and disease, but also showed weaknesses regarding the causal argumentation. This may be the case, for example, (1) if compared with the “positive” studies, there are a considerable number of studies without risk relation, (2) if there is a lack of study results or inconsistent results from intervention studies with intermediary markers, or (3) if meta-analyses gave heterogeneous results. The number of studies required to classify the strength of the evidence as “probable” remains at not <5 very good studies with level of evidence I and/or level of evidence II.

### Possible evidence regarding a preventive effect or a lack of an association

The strength of the evidence was judged as “possible”, if most epidemiological studies, but at least 3, showed consistent results. There may exist a few other studies without any risk relation or with opposite risk relation, respectively.

### Insufficient evidence regarding a preventive effect or a lack of an association

The strength of evidence was judged as “insufficient” if data were lacking because the relation between nutritional factor and disease has not yet or only rarely been investigated in the present studies. Further criteria were inconsistent results with a majority of studies without risk relation and nearly equally as strong opposite results.

However, despite the assignment of the level of evidence to each study and the strict specification of the strength of evidence, the database has not been shown to be always clear. Thus, in addition to the level of evidence and the number of studies, both the assessment of the study quality and the current estimation of the studies’ importance based on its design and size were considered as well.

The evaluation of the strength of evidence does not include the estimate of the quantity of intake of vegetables and fruit. In view of the ranking ability of the food frequency questionnaire as prime measurement feature that is the most often used dietary assessment instrument in cohort studies, and the difficulty in estimating adherence to dietary behaviour in intervention studies, we also abstained from considering the published intake values of vegetables and fruit. In addition, we would like to note that in nearly all of the studies, a linear model of trend across the ordered categories of intake was fitted meaning that a change in intake has been related to risk and not an absolute intake.

## Consumption of vegetables and fruit

For the European Nutrition and Health Report [4], food consumption in Europe was analysed with data from representative nutrition surveys in 19 countries, which were documented in a database of the European Food Safety Authority (as of 2008). Data were directly comparable only to a limited extent due to different survey methodology and periods. However, the following results on the average consumption of vegetables and fruit per person can be derived: along with Poland, Italy, Austria, Germany is among the 4 countries in which an average of more than 400 g vegetables and fruit was consumed daily. The consumption of vegetables in Southern Europe (Greece, Italy, Portugal, Spain, Cyprus) as well as in Central and Eastern Europe (Germany, Austria, Poland, Romania, Slovenia, Czech Republik, Hungary) with about 250 g/day was higher than in Northern Europe (Denmark, Estonia, Finland, Latvia, Lithuania, Norway, Sweden) with 140 g/day. The highest fruit consumption was found in Central and Eastern Europe (209 g/day) as well as in Southern Europe (203 g/day). In Northern Europe, the fruit consumption was 129 g/day and in Western Europe (Belgium and Luxembourg, France, Ireland, The Netherlands, Great Britain) 113 g/day.

This south–north gradient was also observed in a cross-sectional analysis of the consumption data (24-h recall) in 35,955 men and women from the EPIC cohorts in 10 European countries. In men, the highest mean vegetable intake adjusted for age, season, and day of the week was observed in Greece (270 g/day), the lowest (103 g/day) in Umeå (Sweden). In men from Germany, the vegetable intake was 170 g/day (Heidelberg) or 151 g/day (Potsdam). In women, vegetable intake was highest in southern France (261 g/day) and lowest in Asturias, Northern Spain (103 g/day). In the German survey centres, the intake in women was about 165 g/day. The mean fruit intake adjusted for age, season, and day of the week in men was between 454 g/day in Murcia (Spain) and 122 g/day in Malmö (Sweden), and in women between 400 g/day in Ragusa (Italy) and 151 g/day in Malmö (Heidelberg: men 175 g/day and women 213 g/day; Potsdam: men 239 g/day and women 260 g/day) [5].

Data from 196,373 adults from 52 countries with mainly small and middle income who were interviewed in the *World Health Survey* (2002–2003) (24-h recall) showed that about 78 % of the men and women consumed <5 portions of vegetables and fruit daily as recommended by the World Health Organisation (WHO, according to the WHO: 400 g/day) [6].

## Judgement of the evidence regarding individual diseases

In the following, at first, the symptoms of the individual diseases and the most important influencing factors are described. Then, the available data and the most important studies are summarised, and in conclusion, the strength of the evidence is judged.

### Obesity

The prevalence of pre-obesity and obesity^1^ has been rising in recent decades in European countries. For example, in the EPIC–DIOGENES cohort, the prevalence of obesity in 60- to 65-year-olds increased within 8.6 years of follow-up from 21.5 to 27.8 %. In this cohort study, it was also observed that in the current generation of elderly people, overweight persisted into old age once it has been developed [7]. Overweight or obesity occurs disproportionately often in individuals that have unfavourable socioeconomic indicators regarding education, income, and professional position [8]. Particularly, alarming is the sharp increase in obesity in children and adolescents. According to the data of the *PreVENT Study*, which includes the results of the German representative national *KiGGS Study* and also of other large surveys in Germany (*KOPS, IDEFICS, CHILT*), 12 % of the 3- to 6-year-old, 17.9 % of the 7- to 10-year-old, 18.9 % of the 11- to 13-year-old, and 15.0 % of the 14- to 17-year-old children and adolescents are overweight.^2^ Averaged over all age groups, nowadays, 6 % of the children and adolescents are obese^3^ (Müller M, own results).

Overweight occurs if energy intake is higher than energy expenditure. Compared with many other foods, the volume of vegetables and fruit in relation to the energy content is larger. Due to the favourable volume to energy ratio of vegetables, and fruit, satiety signals can emerge without consuming a large amount of energy [9]. The extent is not known to which individual constituents of vegetables and fruit such as dietary fibre are involved in the regulation of hunger and saturation and hence body weight.

The association between vegetable and fruit consumption and weight development was summarised in the *ISAFRUIT Project* of the EU from 2008 [12]. Eleven out of the 16 identified studies observed an inverse association, including 3 intervention studies and 8 prospective observational studies. In addition to the 8 prospective studies of the ISAFRUIT summary, including 5 studies that showed an inverse relation, there are other prospective studies on the association between the consumption of vegetables and fruit and weight change, which either have been published later than the ISAFRUIT summary or have not been included in the summary. They either showed an inverse relation [13–16] or no relation or relations that were only evident in subgroups differentiated by gender or food groups [17–19]. In one of the studies, a positive relation was observed [20]. Some of the studies investigated the consumption of vegetables and fruit in relation to a dietary pattern. In these studies, the role of vegetable and fruit consumption per se is difficult to assess. In longitudinal investigations in infants and children (observation periods were between 1 and 8 years), the consumption of vegetables and fruit did not have a significant influence on the maintenance of normal weight^4^ or the incidence of overweight [21, 22]. Children with persistent overweight throughout the observation period had a higher fat and a lower vegetable and fruit consumption than overweight children, who could reduce weight during the observation period [23]. However, it is not possible to detect differences in the effects of fat and vegetables and fruit in this study. The same weak or not evident influence was seen in results from cross-sectional studies ([24, 25], PreVENT unpublished data). Contradictory, a prospective study showed that a high consumption of fruit juice had a minor positive influence on weight gain [26].

Intervention studies with vegetables and fruit without focus on weight reduction were systematically analysed in a review [27]. The few studies that only had vegetables and fruit as an intervention either showed no changes in weight development or observed weight changes were comparable to the control group. A slightly more favourable effect regarding weight development was observed in studies with simultaneous fat reduction, as in some of these interventions, spontaneous weight loss occurred. Intervention studies on weight reduction are investigations that only indirectly provide information on the role of vegetables and fruit for weight development. Instructions to eat more vegetables and fruit to stabilise weight resulted in variable extents of weight reduction including substantial weight loss. This weight loss had been linked to reduced energy density [28]. It was shown in an intervention study that at fat reduction, an increase in vegetable intake enhances weight loss [29]. However, another intervention study with 1,510 women with breast cancer did not observe weight loss with such an intervention over 4 years [30].

In summary, these studies showed that an increase in vegetable and fruit consumption might be a suitable measure to facilitate initial weight loss and subsequent weight stability [27]. In this context, it seems also to be important to address energy reduction as well. We could not identify studies investigating in children and adolescents, whether an increase in vegetable and fruit consumption influences body weight.

For the range of normal weight and slight overweight, the *Women’s Health Initiative* (WHI) *Dietary Modification Trial* reported about the role of vegetables and fruit for long-term weight stability*.* In this randomised intervention study including 48,385 women (aged 50–79 years), the intervention group was given specific advice regarding an increase in the consumption of both vegetables and fruit (target ≥5 portions/day) and cereal products (target ≥6 portions/day) as well as a reduced intake of fat (target <20 energy %). A first analysis showed a lower weight increase in normal-weight women in the intervention group (+1.1 portions of vegetables and fruit per day at fat reduction of 8 energy %) than in the control group, while in obese women, weight reduction was observed in both, the intervention and the control group. Thus, using all study data and a multivariate regression model, the change in consumption of vegetables and fruit was just not significantly (*p* = 0.06) associated with weight increase, with one portion being associated with 60 g body weight increase over 9 years. Another analysis across all BMI classes showed that this relation is nonlinear and that an increase in consumption of vegetables and fruit of more than 2.2 portions per day (ca. 200 g) was associated with weight reduction [31]. As the primary objective of this multiple intervention was a reduction in fat intake, the significance of this study regarding the benefit of vegetable and fruit consumption is limited.

It can be concluded from both the prospective and the intervention studies that there is *possible* evidence that an increase in the consumption of vegetables and fruit contributes to *weight stability* (i.e. no weight increase occurs). There is also *probable* evidence that an increase in vegetable and fruit consumption alone does *not* result in *weight loss*. There is *probable* evidence that an increase in the consumption of vegetables and fruit leads to weight reduction, if this replaces foods rich in fat or energy. In children and adolescents, there is only *insufficient* evidence regarding an association between the consumption of vegetables and fruit and *weight development* due to a lack of intervention studies and the existence of only a few cohort studies with no risk relation.

### Type 2 diabetes mellitus

Type 2 diabetes mellitus is one of the most common and most expensive chronic diseases. According to the International Diabetes Federation, the diabetes prevalence in the 20- to 79-year-olds was 6.4 % for women [32] with large regional differences (e.g. 3.8 % in Africa, 6.9 % in Europe, and 10.2 % in North America). Due to ageing of populations, this prevalence is expected to increase to 7.7 % by the year 2030 with an expected 237 million affected individuals. These estimates include millions of undetected cases, because at the beginning, the disease often is free of symptoms and is only diagnosed years later [33], but does not include the rise of prevalence due to changes in other major risk factors beyond age, like the rise of obesity prevalence rates and adoption of Westernised diet and lifestyle habits in many parts of the world. The prognosis of affected individuals is crucially determined by the presence of accompanying risk factors and by the development of micro- and macroangiopathic complications. Cardiovascular events like myocardial infarction, stroke, and peripheral arterial circulation disorders are predominant [34].

Type 2 diabetes mellitus develops due to a complex interaction between genetic predisposition and lifestyle. The actual manifestation of the disease is preceded by a phase of impaired glucose regulation, in which the cardiovascular risk is already increased. Particularly important among the lifestyle factors that promote or accelerate the manifestation of type 2 diabetes mellitus are bad nutritional habits and a lack of physical activity [35]. However, the most important risk factor for the development of type 2 diabetes mellitus is truncal obesity, which also is the result of an unfavourable lifestyle including overeating and a lack of physical activity.

The results of several prospective cohort studies that investigated whether the consumption of vegetables and fruit is associated with the risk of type 2 diabetes mellitus were summarised in 2 meta-analyses. The meta-analysis by Hamer and Chida [36] including 5 cohort studies in total did not show a relation between the consumption of fruit and/or vegetables and the risk of diabetes. Individuals who consumed at least 5 portions of vegetables and fruit per day had a relative risk (RR) of 0.96 (95 % CI 0.79–1.17) compared with persons with low consumption (lowest quintile or non-consumers; 3 cohort studies). For vegetables and fruit analysed separately (4 cohort studies each), there also was no association (RR regarding ≥3 vs. <3 portions/day: fruit consumption: 1.01; 95 % CI 0.88–1.15; vegetable consumption: 0.97; 95 % CI 0.86–1.10). In another meta-analysis [37], 2 more recent cohort studies were included, but one study that was included into the meta-analysis by Hamer and Chida was not considered. Here again, there was no risk relation regarding the total intake of vegetables and fruit: the RR for the comparison of the highest with the lowest category of consumption was 1.00 (95 % CI 0.92–1.09). Also, the consumption of either fruit (RR 0.93; 95 % CI 0.83–1.01) or vegetables alone (RR 0.91; 95 % CI 0.76–1.09) was not associated with the risk. However, the risk of diabetes was significantly reduced in persons that consumed relatively large amounts of green leafy vegetables. Other subgroups of vegetables and fruit have not been investigated.

In addition to the studies considered in the meta-analyses, some other prospective cohort studies exist, but in general, they also did not observe a significant relation between the overall consumption of vegetables and fruit and the risk of diabetes [38–40]. However, in the *EPIC*-*Norfolk Study* [40], a significant risk reduction was observed with increased fruit consumption (RR for the comparison of highest and lowest quintile: 0.70; 95 % CI 0.54–0.90). In a meta-analysis of cohort studies, no significant associations were observed between the intake of dietary fibre from fruit (9 individual cohort studies; RR comparing extreme quintiles/quartiles 0.96; 95 % CI 0.88–1.04) or vegetables (7 individual cohort studies; RR comparing extreme quintiles/quartiles 1.04; 95 % CI 0.94–1.15) and the risk of diabetes [41].

The present cohort studies were usually adjusted for BMI, as the possible effect of a higher vegetable and fruit consumption on body weight could not be separated from the potential confounding effect of body weight. Therefore, the results of the cohort studies describe the relation between vegetable and fruit consumption and the risk of diabetes excluding this important factor, through which the consumption can ultimately influence the risk of diabetes. In randomised controlled intervention studies, it was shown that a change in lifestyle with a focus on weight reduction through dietary changes can reduce the conversion from impaired glucose tolerance to type 2 diabetes [42–44]. However, the role of vegetable and fruit consumption remained unclear in these studies, as the interventions were designed multifactorially and included increased physical activity in addition to dietary changes [43, 44]. It may still be expected that higher consumption of vegetables and fruit can lower the risk of diabetes, as such a dietary change might prevent the development of obesity ([27], see “Obesity”). In the intervention arm of the *WHI Dietary Modification Trial* (see “Obesity”), an increase in vegetable and fruit consumption by 1 portion combined with a reduction in the fat proportion by 8 % of energy intake did not result in a changed risk of type 2 diabetes mellitus over 7 years [45].

In summary, it can be concluded that most of the studies and their meta-analysis indicate a lack of an association between the consumption of vegetables and fruit and the risk of diabetes. Because of this, there is *probable* evidence that the risk of developing type 2 diabetes mellitus is not influenced by the consumption of vegetables and fruit. However, vegetables and fruit indirectly influence the prevention of type 2 diabetes mellitus, as consumption thereof might lower the risk of weight gain in adults.

### Hypertension

Hypertension is one of the most relevant clinical findings for public health policy, with a global prevalence of 26 % in the adult population in 2000. Twenty-nine percent were projected to have this condition by 2025 [46]. About 90 % of subjects with hypertension suffer from essential hypertension, that is, hypertension is not the consequence of another disease. Due to the increased risks of stroke and CHD [47], and also of renal cancer [48] associated with hypertension, lifelong medication is usually required. It could be shown that even a slight reduction in the mean blood pressure in the population strongly reduces the incidence of cardiovascular diseases [49, 50]. The *American Heart, Lung and Blood Institute* has stated in 2003 that the measures for the prevention of hypertension include a health-promoting lifestyle which in addition to weight reduction (at existing overweight) comprise the adherence to the DASH diet,^5^ the limitation of sodium and alcohol intake as well as increased physical activity [51]. The *ESH*–*ESC Task Force on the Management of Arterial Hypertension* [52] of the European Society of Hypertension regards the increase in the consumption of vegetables and fruit as one of the lifestyle measures that can lower blood pressure in individuals with only a few risk factors for cardiovascular diseases and slightly increased blood pressure.

The *INTERSALT Study* with data of more than 10,000 subjects from 52 centres in 32 countries has shown an inverse relation between the intake of potassium (a mineral associated with a plant-based diet high in vegetables and fruit) and blood pressure, independent of the quantity of sodium intake [53]. However, in a small tightly controlled intervention study over 6 weeks including 48 participants with slightly increased blood pressure, such an effect could not be shown [54].

In vegetarians, there is often a lower blood pressure observed than in the total population, and a reduction in the blood pressure has been seen after changing from a normal to a vegetarian diet [55]. In cohort studies, there were either inverse relations between the consumption of vegetables and fruit and new cases of hypertension [56, 57] or inverse relations with one of the two food groups considered here or with a dietary pattern including vegetables and fruit [58, 59]. In the cross-sectional and in the longitudinal analysis of the *SU.VI.MAX Study,* an inverse relation was observed between vegetable and fruit consumption and blood pressure [60]. There was no relation seen regarding other components of the DASH diet. The intervention with antioxidant vitamins did also not influence the development of blood pressure. In the *SUN cohort study* in turn, it was observed that a high consumption of vegetables and fruit was only associated with a reduced risk of hypertension, if the consumption of olive oil was low (<15 g/day) [61]. Another analysis of the *Nurses’ Health Study* (NHS) I and II and the *Health Professionals Follow*-*up Study* (HPFS) after 14 years of follow-up with flavonoid intake calculated by an updated nutrient database from 2010 showed a risk reduction in hypertension with increasing intake of anthocyanins [62].

The DASH diet is based upon the *DASH Study*, which is a randomised 8-week intervention study including 459 hypertensive patients. One intervention group was instructed to eat a diet rich in vegetables and fruit, and the other group got the same instructions with additional information on a diet low in fat and high in dietary fibre. In both intervention groups, a lowering of blood pressure was reported [63]. In the latter group, the blood-pressure-lowering effect was more pronounced than in the group that was only instructed to eat a diet rich in vegetables and fruit. Other intervention studies have confirmed the effectiveness of the DASH diet as measure for reducing blood pressure levels. For example, the DASH intervention in the *Premier Trial Study* including 810 adults with hypertension achieved a greater decrease in blood pressure levels by an increased consumption of vegetables and fruit as well as of low-fat dairy products than the intervention with weight reduction, enhanced physical activity, and limitation of sodium intake [64]. In children and adolescents, too, this diet is suitable to lower blood pressure levels [65]. A 6-month intervention study including 690 subjects at the age of 25–64 years in England confirmed the results of the DASH study [66]. In this study, an increase in the consumption of vegetables and fruit to at least 5 portions/day was accompanied by a lowered blood pressure. Furthermore, the study showed that an increase in vegetable and fruit consumption does neither lower the blood cholesterol concentration nor leads to weight loss, but keeps weight stable. An intervention study conducted in the 1990s including 78 participants with low consumption of vegetables and fruit (<3 portions/day) revealed that lipid and lipoprotein metabolism are not influenced by an increase in vegetable and fruit consumption [67].

Based on the present data, the evidence regarding a *blood*-*pressure-lowering effect* of an increase in the consumption of vegetables and fruit is judged as *convincing*. Both cohort and intervention studies show consistent results.

### Coronary heart disease

Coronary heart disease (CHD) is the most important manifestation of arteriosclerosis in humans and belongs to the large group of cardiovascular diseases. CHD is still the single largest cause of premature death in the world. Ischaemic heart disease has been estimated to account for 12 % of all deaths worldwide in 2004 [68]. In 2008, 17 million deaths worldwide were due to cardiovascular diseases, accounting for 48 % of non-communicable disease deaths [69]. While CHD death rates have declined in many parts of the industrialised world, death rates are increasing in most developing countries [70]. CHD is also a major cause of disease burden in terms of disability-adjusted life years lost (DALY), accounting for 63 million DALYs worldwide in 2004 [68].

In addition to age and gender, modifiable risk factors are important, especially lifestyle factors like smoking and a lack of physical activity and the medical diagnoses hypertension, diabetes mellitus, obesity, and dyslipoproteinaemia [71]. Among these factors, the 4 medical diagnoses are clearly nutrition-related and can be influenced by a change in nutrition. Other biological mechanisms that are probably important in atherogenesis are influenced by nutrition, including inflammatory processes, oxidative stress, and increased homocysteine concentrations [72].

Several prospective cohort studies that investigated whether the consumption of vegetables and fruit is associated with the risk of CHD were summarised in 2 meta-analyses. In the meta-analysis by Dauchet et al. [73], which included 9 cohort studies, the risk of CHD was reduced by 4 % (RR 0.96; 95 % CI 0.93–0.99) per portion of vegetables and fruit and by 7 % (RR 0,93; 95 % CI 0.89–0.96) per portion of fruit daily. For vegetables, the inverse relation regarding the risk of CHD was stronger for the overall cardiovascular mortality (RR per portion 0.74; 95 % CI 0.75–0.84) than for fatal or non-fatal myocardial infarction (RR 0.95; 95 % CI 0.92–0.99). Between mortality and consumption of fruit as well as total intake of vegetables and fruit, a linear dose–response relation was observed. In contrast, the relation between mortality and consumption of vegetables was nonlinear. The meta-analysis of He et al. [74] included 13 cohort studies. Compared with individuals who consumed <3 portions of vegetables and fruit per day, persons with a consumption of 3–5 portions per day (RR 0.93; 95 % CI 0.86–1.00) and of >5 portions per day (RR 0.83; 95 % CI 0.77–0.89) had a lower risk of CHD. Subanalyses revealed a significant inverse relation with the risk of CHD both for fruit and for vegetables. In the following years, after the publication of these meta-analyses, the result of other cohort studies was published. A higher vegetable and fruit intake was inversely associated with the risk of CHD in the *EPIC*-*Heart Study* [75], the *Morgen Study* [76], a Swedish [77], and a Japanese cohort [78], while in the Italian arm of the EPIC Study, no association was found for vegetables and fruit in total, but for leafy vegetables [79]. These data are also reflected in the judgement of the WHO [80] and current nutritional recommendations of the *European Society of Cardiology* [71] and the *American Heart Association* [81] that both recommend the consumption of vegetables and fruit to reduce the risk of CHD.

However, the results of the *WHI* *Dietary Modification Trial* (see “Obesity”) suggest that an additional portion of vegetables and fruit daily does not influence the risk of CHD [82]. As the primary objective of this multiple intervention is a reduction in fat intake, the significance of this study regarding the assessment of the benefit of vegetable and fruit consumption is limited.

The data on the outcome “CHD” are supplemented by intervention studies that have investigated intermediary clinical markers of the cardiovascular system when offering specific kinds of vegetables and fruit. These studies showed that the consumption of vegetables and fruit can improve the regulation of blood vessel enlargement [83], prevent platelet aggregation [84–86], and reduce inflammation markers [87, 88].

In summary, it can be concluded that many cohort studies on this question have been performed, and most of the cohort studies have shown a protective association between the consumption of vegetables and fruit and the risk of CHD. In addition, there are intervention studies that prove a beneficial influence of vegetables and fruit on metabolic pathways that are associated with the risk of CHD. Therefore, the evidence regarding the prevention of CHD by high consumption of vegetables and fruit is judged as *convincing*.

### Stroke

Stroke is one of the major causes of death in the world. In 2004, 9.7 % of all deaths were caused by stroke [68]. Stroke causes also a considerably proportion of disability adjusted life years (DALYs), ranking sixth among the leading causes worldwide [68].

In addition to age and gender, modifiable risk factors are important, especially lifestyle factors like smoking and a lack of physical activity as well as postmenopausal hormone replacement therapy, and the diagnoses hypertension, diabetes mellitus, obesity, dyslipoproteinaemia, CHD, arterial occlusive disease, extracranial stenoses, or occlusion of the arteries supplying the brain [89]. The clinical findings of these factors are clearly nutrition-related and can be influenced by a change in nutrition.

The results of several prospective cohort studies that investigated whether the consumption of vegetables and fruit is associated with the risk of stroke were summarised in 2 meta-analyses [90, 91]. In the first meta-analysis, including 7 cohort studies, the risk of stroke was reduced by 11 % (RR 0.89; 95 % CI 0.85–0.93) per portion of fruit per day, by 5 % (RR 0.95; 95 % CI 0.92–0.97) for vegetables and fruit, and by 3 % (RR 0.97; 95 % CI 0.92–1.02) for vegetables [90]. In this meta-analysis, a linear dose–response relation was observed. The second meta-analysis included 9 individual cohort studies [91]. Compared with individuals with an intake of vegetables and fruit of <3 portions per day, subjects with 3–5 portions per day (RR 0.89; 95 % CI 0.83–0.97) and with >5 portions per day (RR 0.74; 95 % CI 0.69–0.79) had a significantly lower risk of stroke. These results were confirmed by a study that was published after the meta-analyses. In this cohort study with Japanese participants, a higher fruit consumption was associated with a significantly lower risk of stroke (RR for the comparison of the highest with the lowest quintile of consumption: 0.67; 95 % CI 0.55–0.81) [78]. However, there was no significant relation between the intake of vegetables and the risk of stroke. Overall, the available data indicate a risk-reducing effect of vegetable and fruit consumption. This is also reflected in the judgement of the WHO [80] and current dietary recommendations of the *European Society of Cardiology* [71] and the *American Heart Association* [89].

In the *WHI Dietary Modification Trial* (see “Obesity”), with an additional portion of vegetables and fruit per day, there was no difference regarding the occurrence of stroke [82]. However, the significance of this study is limited, because the primary objective of this multiple intervention was a reduction in fat intake.

The data on the outcome “stroke” are supplemented by intervention studies that have investigated intermediary clinical markers of the cardiovascular system when offering specific kinds of vegetables and fruit (see “Coronary heart disease”; [83–88]).

The meta-analyses of cohort studies clearly indicate that there is an inverse association between the consumption of vegetables and fruit and the risk of stroke. Additional intervention studies show a favourable influence of the consumption of vegetables and fruit on important metabolic pathways, which also have an impact on the risk of stroke. From these results it can be concluded that a high intake of vegetables and fruit reduces the risk of stroke with *convincing* evidence.

### Cancer

In 2008, about 2,457,610 new cases of cancer were observed in the European Union [92]. For the same year, cancer was recorded as cause in 1,231,220 deaths. Therefore, both in numerical and in socioeconomic terms, cancer is one of the most important chronic diseases in the European Union.

The occurrence of cancer as a whole is increasing with age and the pathogenesis often takes several decades. The disease is characterised by chromosomal changes that can be induced due to different reasons. In addition to age, the most important risk factors include tobacco smoking, consumption of alcohol, overweight, hormonal factors, physical activity, and food intake [2].

A summary published in 1992 of the results of epidemiological studies, mostly case–control studies, on the association between consumption of vegetables and fruit and the occurrence of cancer showed high consistency regarding an inverse risk relation (128 out of 156 studies; [93]). This resulted in the “5 a day” campaign in the USA with the aim to reduce the incidence of cancer. In the report of WCRF experts published in 1997, which was based upon data until the beginning of the 1990s, vegetables and fruit were rated among the most important cancer preventive factors with a calculated prevention potential of 23 % and the strength of evidence was rated as convincing for many cancer sites [94]. Similar, but also lower prevention figures were revealed for some European countries when using a different methodological approach and similar relative risk estimates [95, 96].

In 2003, a new revaluation of the cancer preventive potential of vegetables and fruits was performed by an expert panel of the International Agency for the Research on Cancer [97]. As data from prospective cohort studies had become available increasingly, they were included in this evaluation for the first time. This new evaluation resulted in strength of the evidence that was one grade lower than in the WCRF report. According to the data in 2003, there was probable evidence for a protective effect of vegetables regarding cancer of the oesophagus and colon and rectum, and possible evidence regarding cancer of the oral cavity, pharynx, stomach, larynx, lung, ovary and kidney. There was probable evidence for a protective effect of fruit regarding cancer of the oesophagus, stomach, and lung and possible evidence for a protective effect regarding cancer of the oral cavity, pharynx, colon, rectum, larynx, kidney, and bladder. A meta-analysis published at the same time period resulted in the same conclusions [98]. The data available until 2007 and a detailed systematic evaluation of the evidence regarding the different sites of cancer are included in the German Nutrition Report 2008 [99]. This evaluation will be continued in the German Nutrition Report 2012.

Currently, data are dominated by the results of the large prospective cohort studies such as *EPIC* [100] and *NIH–AARP Study* [101], each including more than 500,000 participants, and the *Pooling Project,* which is a pooled analysis of up to 17 cohort studies. Key [102] summarised the results of these studies until 2009, both for cancer in general and regarding the most important cancer sites. The data regarding the different cancer sites are characterised by reduced risks in connection with high consumption of vegetables and fruit; however, the risk relations are often not statistically significant or only just significant, and the risks differ depending on the smoking behaviour. Therefore, the data situation regarding a specific cancer site appears to have a high degree of complexity, and conclusions for a specific cancer site cannot be drawn within the context of this review. Regarding a judgement on the association between the consumption of vegetables and fruit and different types of cancer, we therefore refer to future work.

Several studies have investigated the relation between the consumption of vegetables and fruit and cancer in general [103–106]. Such investigations do not provide information on specific mechanisms, but are important for public health, as they give an overall evaluation. The analyses of the Harvard studies (NHS I, NHS II, HPFS) and of a Japanese study did not indicate a relation between this nutritional factor and the risk of cancer [103, 104]. The analysis of the NIH–AARP showed a significantly reduced risk at high vegetable intake in men, but not in women [105]. In the *EPIC study*, a lowered risk of cancer was observed both with higher intake of vegetables and with higher intake of fruit [106]. In all of the studies, the reduction in risk was small in view of the investigated range of consumption. In addition, it has to be considered that the risk reduction was mainly seen in those types of cancer that are associated with smoking [106]. Therefore, it remains unclear, whether this risk reduction goes along with a lifestyle of high exposure to carcinogens, or whether the risk reduction is due to a lack of statistical control of the smoking factor.

In addition to cardiovascular diseases, the aim of the *WHI* *Dietary Modification Trial* (see “Obesity”) was the investigation into colon and breast cancer. Compared with the control group, the achievements in the intervention arm of an increase in vegetable and fruit consumption by 1 portion per day and a reduction in the percentage of fat on energy intake by 8 % did not result in a changed risk of colon cancer over 7 years and resulted in only a slight, non-significant reduction in risk of breast cancer [107, 108]. Although the significance of this study regarding vegetables and fruit is limited due to the multiple interventions, the results are in accordance with the results obtained from the observational studies by confirming that there will be no detectable effects on risk of cancer if there are only small differences in the consumption of vegetables and fruit.

The risk reductions that have been observed in some large cohort studies with increasing consumption of vegetables and fruit still suggest that the consumption of vegetables and fruit influences the risk of cancer. However, this influence is only detectable if there are large differences in the consumption of vegetables and fruit between the groups and could appear only in case of high exposure to carcinogens, like, for example, in smokers. However, these restrictive statements do not directly influence the evidence regarding an inverse relation between the consumption of vegetables and fruit and the risk of cancer, which is judged as *probable*.

### Chronic inflammatory bowel diseases

Chronic inflammatory bowel diseases (IBD) like Crohn’s disease and ulcerative colitis are examples of chronically recurrent diseases of the gastrointestinal tract. Incidence and prevalence of these diseases are increasing in Western industrial countries [109, 110]. Both disorders affect people in approximately equal female/male proportion with a combined mean frequency of 5–200 cases per 100,000 European and North American inhabitants [110]. The incidence of Crohn’s disease is still increasing in Western societies, demonstrating the importance to add mechanistic insights into the yet unknown aetiology of the disease pathogenesis. The low concordance rate in identical twins for Crohn’s disease (~50 %) and ulcerative colitis (~10 %) confirms epidemiologic observations that environmental factors strongly contribute to the disease progression [111].

The aetiology of Crohn’s disease and ulcerative colitis is still not known, but evidence is growing that environmental factors (nutrition, smoking, infections) combined with genetic predisposition strongly enhance the risk of this disease [112]. The clinically manifest inflammation of the disease might be caused by a primary intestinal barrier malfunction that leads to a secondary inflammatory response and is maintained by chronically activated immune cells in the bowel [113]. The results of many clinical and experimental investigations into gnotobiotic animal models in recent years show that an imbalance between intestinal microorganisms (microbiome) and the immune system contributes significantly to the development of chronic inflammatory processes in the bowel [114]. These uncontrolled activation reactions in the bowel cause tissue damage that affects all layers of the intestinal wall in Crohn’s disease (transmural), whereas in ulcerative colitis, it mainly involves the superficial epithelial layer of the colon. Recent animal studies show the influence of iron on the composition of the intestinal microbiome and that a high intake might be involved in the pathogenesis of chronic inflammatory processes in the bowel [115]. In addition, the role of vitamin D deficiency in the pathogenesis of chronic intestinal inflammatory processes and the IBD-associated colorectal carcinoma is also discussed [116]. The close interaction of the composition and function of the microbial ecosystem with nutritional factors suggests that the intake of vegetables and fruit might be involved in the occurrence of inflammatory processes in the bowel [117].

A first systematic review (5 case–control studies on Crohn’s disease and 8 case–control studies on ulcerative colitis) concluded that a high intake of fruit is associated with reduced risk of Crohn’s disease; there was no statistically significant association regarding vegetables. No association was found between ulcerative colitis and fruit, while a trend was observed towards a risk reduction regarding vegetables [118]. There are no prospective cohort and intervention studies that investigated the role of vegetables and fruit for the aetiology of IBDs.

There is *insufficient* evidence regarding the association between the consumption of vegetables and fruit and the risk of developing IBDs.

### Rheumatoid arthritis

Rheumatoid arthritis (RA) is the most common rheumatic disease. In industrialised countries, 0.3–1.5 % of the population suffers from RA [119]. Women are affected three times more often than men [120]. RA is a chronic inflammatory disease that primarily affects the joints. The cause of the disease is unknown to a large extent. In addition to genetic factors, smoking, overweight, and nutrition contribute to the risk of disease [121]. As to nutrition, the risk seems to be increased by the consumption of red meat, protein, and coffee, while it is lowered by oily fish and olive oil.

To estimate the importance of the consumption of vegetables and fruit for the development of RA, 4 prospective cohort studies [121–124], 1 cross-sectional study [125], 1 case–control study [126], and 1 intervention study [127] in total were identified. Most of the cohort studies show a reduced risk at high consumption of vegetables and fruit [121–123]. The study that did not find an inverse association did not report the absolute amount of vegetable and fruit consumption [24]. Therefore, it is difficult to compare this study with the other cohort studies. In the only available case–control study, higher consumption of cooked vegetables (2.9 servings/day) was significantly associated with lower risk, while raw vegetables were not effective [126]. In a cross-sectional study by Wang et al. [125], less bone marrow lesions were observed in healthy individuals with high intake of fruit. Vegetable intake was not significantly associated with bone measures. In a pilot study in women suffering from RA, a long-lasting improvement of symptoms was achieved through the intervention resulting in a small increase in intake of fruit, vegetables, and legumes (increase from 3.4 to 3.7 total servings/day) [127].

The evidence regarding the prevention of RA with a high intake of vegetables and fruit is judged as *possible* due to the low number of studies.

### Chronic obstructive pulmonary disease

The pooled prevalence for chronic obstructive pulmonary disease (COPD) for 28 countries has been reported to be 7.6 %, and for adults aged ≥40 years, the prevalence is 9 %-10 % [128]. The disease is associated with narrowing (obstruction) of the airways, which causes typical breathing sounds such as whistling or wheezing. According to estimates of the WHO, by the year 2020, COPD will be the third most common cause of death worldwide. The diagnosis of COPD is confirmed by a test that measures the forced expiratory volume in 1 s (FEV1), which is the greatest volume of air that can be breathed out in the first second of a large breath. A high FEV1 value indicates normal pulmonary function. Smoking is the most important risk factor of COPD.

A total of 22 studies were analysed. Four of the studies were prospective cohort studies [129–132] and 2 were case–control studies [133, 134], while the majority were cross-sectional studies [135–150]. In the prospective cohort studies by Miedema et al. [129], the consumption of fruit was inversely associated with the risk of COPD (RR 0.68; <14 g/day vs. >70 g/day). In the prospective study by Walda et al. [130], an increase in fruit consumption by 100 g/day was associated with a reduction in the COPD risk by 24 %. In both studies, no association was found between the risk of COPD and vegetable intake. In the HPFS and the NHS, Varraso et al. [131, 132] investigated dietary patterns and observed a risk of COPD lowered by up to 50 % with a diet high in fruit, vegetables, and fish. The case–control study by Hirayama et al. [134] determined a significantly lower intake of vegetables and fruit in COPD patients than in control persons. There was a primary inverse correlation between the prevalence of COPD and the quantity of vegetable intake. In the second case–control study in smokers, high consumption of vegetables (≥93 g/day) and fruit (≥121 g/day) was associated with a COPD risk reduction by 54 % each. High intake of apples (≥3 apples/week) resulted in a reduction in the COPD risk by 53 % [133].

Most of the cross-sectional studies also show a significant positive association between the quantity of fruit intake and the FEV1 or reduced occurrence of obstruction, respectively [135–138, 140–144, 146, 147, 149, 150]. Only 3 studies indicate a risk reduction due to high consumption of vegetables [144, 145, 148]. In one study, for both the intake of flavonoids and the consumption of apples and pears, a significant positive association with the FEV1 value was found [141, 143]. The intake of dietary fibre from fruit was also associated with a reduced risk of COPD [151, 152]. As only a few cohort studies exist and there are mainly cross-sectional studies, the evidence regarding the association between high intake of vegetables and fruit and reduced risk of COPD is currently judged as *possible*.

### Asthma

Asthma is one of the most common chronic diseases and occurs in 5–10 % of the population in Western countries [153]. In addition to genetic factors, environmental factors including nutrition are primarily responsible for the increase in the prevalence of asthma in recent decades [154]. Asthma is often accompanied by increased sensitivity to allergies. Various nutritional factors (like oily fish, unsaturated fatty acids, vitamins, and micronutrients) probably influence the risk of asthma [155, 156].

For judging the evidence regarding the association between risk of asthma and consumption of vegetables and fruit, a total of 20 studies were identified (adults and children at the age of ≥4 years), 10 of which were cross-sectional studies [157–166], 4 were case–control studies [167–170], 4 were cohort studies [138, 171–173], and 1 was an intervention study [174]. All studies except of those by Huang et al. [158], Garcia et al. [168], and Lewis et al. [162] showed an inverse association between the incidence of asthma and the intake of fruit or of vegetables and fruit, respectively. This association is particularly obvious for apples [160, 161, 167, 171]. In the cohort study by Willers et al. [172], apples also were identified as food that is associated with a reduced risk of asthma. A high consumption of apples in pregnant women was accompanied by a lower risk of asthma in the children within the first 5 years after birth. In another cross-sectional study, the consumption of apple juice, but not of fresh apples was inversely associated with the risk of asthma [175].

In an intervention study with asthma patients, a control diet restricted in the intake of vegetables and fruit enhanced the asthma symptoms, while the supplementation of tomato juice improved the symptoms [174].

Only in one cohort study [171] and one cross-sectional study [161], the consumption of vegetables alone was inversely associated with the risk of asthma. Preliminary results suggest that genetic polymorphisms (mutations in the catalase gene) exert an additional influence on the association between the risk of asthma and protective effects of high vegetable and fruit intake [176].

The available data are mainly based on cross-sectional studies and show consistently that a high fruit and vegetable intake lowers the risk of asthma. Therefore, there is *possible* evidence regarding a protective effect of the consumption of this food group. In this respect, the consumption of fruit seems to be more important than the consumption of vegetables.

### Osteoporosis

Osteoporosis is defined as a skeletal disorder characterised by compromised bone strength predisposing to an increased risk of fracture [177]. Worldwide, an osteoporotic fracture is estimated to occur every 3 s, a vertebral fracture every 22 s [178]. In Europe, estimated 179,000 men and 611,000 women will suffer a hip fracture each year [179], and the lifetime risk of an osteoporotic fracture is up to 53 % in women and up to 22 % in men [180]. Overall, 61 % of osteoporotic fractures occur in women, with a female-to-male ratio of 1.6. Sex-specific fracture rates vary with fracture site: 58, 70, 75, 80 % of spine, hip, humerus, and forearm fractures, respectively, occur in women [178]. Although more women then men are affected, the disease burden in men is considerable: approximately one-third of hip fractures occur in men [181], and their mortality is higher than in women, with about 37.5 % dying within the first 12 months as compared to 28.2 % of the affected women [182, 183].

The after-effects of osteoporotic fractures are severe and include reduced mobility, chronic pain, loss of independence, need for permanent care, and death. In Europe, disability due to osteoporosis is greater than that caused by cancers [178].

Cost estimates for all osteoporotic fractures in Europe amount to €25 billion [179]. Due to the expected demographic changes in Europe, direct health care costs associated with osteoporotic fractures are expected to rise up to €76.8 billion in the year 2050 [184].

In addition to age and sex, established lifestyle-related risk factors of osteoporosis comprise reduced physical activity [185, 186] and the amount of calcium and vitamin D intake [187]. Body mass or body composition parameters, respectively, are putative risk factors [188, 189]. Other nutrition-related risk factors currently under scientific evaluation include the intake of animal and plant proteins, table salt, phytoestrogens, foods like soya or prunes, other vitamins, minerals and phytochemicals as well as the acid–base-balance and the consumption of vegetables and fruit [190–195]. As a potential biological explanation for the effect of vegetables and fruit on bone health, their influence on the acid–base-balance is considered. The latter exhibits putative interactions with bone metabolism [196–199].

Studies assessing the influence of vegetable and fruit consumption on bone health and osteoporosis cover a broad spectrum of topics, since different endpoints are considered. In addition to the clinical diagnosis of osteoporosis or an osteoporotic fracture (direct evidence regarding the effect of vegetable and fruit intake on osteoporosis), changes in bone density and various parameters of bone metabolism (indirect evidence regarding the effect of vegetable and fruit intake on osteoporosis) are investigated.

Firstly, for this review, the available direct evidence was searched for. One systematic review and 4 additional prospective studies were identified that investigated the association between vegetable and fruit consumption and the occurrence of osteoporosis or an osteoporotic fracture. The systematic review included studies that have investigated the influence of vegetables and fruit consumption on bone health in women ≥45 years of age [200]. Observational and experimental studies on the incidence of osteoporotic fractures, on bone density, and on parameters of bone metabolism were taken into account. Four of the 8 studies that were analysed in detail revealed a high risk of bias. The other 4 studies provided no consistently significant indications on a protective effect of vegetables and fruit. The cross-sectional studies showed positive associations between the consumption of vegetables and fruit and bone density in various locations. However, no significant effects were shown by either cohort or intervention studies. Due to the low number of available studies on fracture risk, no separate conclusions regarding this topic were drawn by this review.

In the *WHI Dietary Modification Trial* (see “Obesity”), a slightly reduced risk of falling and a slight decrease in bone density, but no influence on the risk of osteoporotic fractures, were seen in the intervention group after 8 years of follow-up [201]. However, due to the complex intervention in that study, the observed effect is not solely attributable to the consumption of vegetables and fruit.

Based on data from 5 European countries of the *EPIC study*, the incidence of femoral neck fractures was determined over the period of 8 years and examined with respect to associations with the consumption of certain food groups [202]. Among the 18,545 women and 10,538 men aged 60 years and older, 275 femoral neck fractures occurred during follow-up. In multivariate adjusted regression models, no significant association was observed for any of the investigated food groups, including vegetables and fruit. Marginally significant protective effects were shown regarding the consumption of vegetables (HR 0.93; 95 % CI 0.85–1.01).

A Japanese cohort study investigated the association between dietary patterns and fall-related fractures in a group of 877 persons at the age of >70 years. Three dietary patterns were determined. Of these, the “meat pattern” was associated with a lowered risk of fracture and the “vegetable pattern” with an increased risk of fracture [203].

In addition to these studies on direct evidence, selected results of studies on indirect evidence are presented in the following.

In a systematic review, Papaioannou et al. [204] searched for risk factors of low bone mineral density (BMD) in men aged ≥50 years. Neither vegetable nor fruit consumption were found to be risk factors. In a randomised clinical trial regarding the effect of citrate supplementation on parameters of bone metabolism (bone turnover markers) and BMD, the effect of an increase in the consumption of vegetables and fruit by 300 g/day was investigated as additional treatment group. No significant influence on the investigated bone parameters was shown. However, the degree of compliance in that study could not be determined [205]. Using retrospective analyses of data of the *Canadian Multicenter Osteoporosis Study*, the associations between dietary patterns and the occurrence of fractures were investigated [206]. 3,539 women and 1,649 men aged ≥50 years were followed for 10 years with respect to incident fractures. The analysis revealed 2 dietary patterns that were associated with the occurrence of fractures: the pattern “nutrient dense”, characterised by a high consumption of vegetables and fruit, was associated with a reduced fracture risk in women. In men, a similar effect was observed that did not reach statistical significance. Other indirect evidence is derived from a randomised study that investigated the effect of the DASH diet, which includes a high vegetable and fruit intake ([63]; see “Hypertension”), on various markers of bone and calcium metabolism. Compared to the control diet, the DASH diet achieved a significant reduction in bone remodelling [207] in the 186 study participants (age 23–76 years). However, due to the complex dietary intervention, it is not possible to determine which of the changed nutritional factors are responsible for the observed effects. Kaptoge et al. [208] did not find a significant association between the consumption of vegetables and/or fruit and the rate of bone density decreases over a period of 3 years. The study, conducted in England, included 470 men and women between the age of 69 and 79 years. Analyses of the *Framingham Osteoporosis Study* showed that in men (aged 69–97 years), but not in women, a significantly lower decrease in bone density was observed with high consumption of vegetables and fruit over a period of 4 years [209]. Similar results were found using prospective analyses of the *Framingham Heart Study*. Again, a protective effect of high vegetable and fruit consumption was only found in men (aged 69–97 years) [210].

Another aspect investigated is the potential effect of vegetables and fruit consumption during childhood. A prospective study showed that the consumption of vegetables and fruit in compliance with recommendations was an independent predictor of the bone mineral content in boys, but not in girls [211]. A further prospective study found a significantly higher bone mass in children with a high consumption of dark-green and deep-yellow vegetables [212]. DeBar et al. [213] conducted a randomised study over 2 years, in which 228 adolescent girls (in the age between 14 and 16 years) were asked to increase their physical activity and to improve their diet, including an increased consumption of vegetables and fruit. Compared with the control group, the girls in the intervention group had a significantly higher bone density at the spine and the femoral neck. However, due to the complex intervention, the observed effect cannot be attributed to the consumption of vegetables and fruit only.

Another interesting aspect considered in various studies is the maternal dietary influence before and during pregnancy on parameters of bone health in children. For example, dietary patterns during pregnancy were associated with the bone density and bone mineral content of the children at the age of 9 years. This long-term study showed that a maternal diet with a high proportion of vegetables, fruit, and whole-grain products was associated with significantly higher levels of bone density and bone mineral content in the offspring [214]. Another study from India showed that a maternal diet with foods rich in calcium, including green leafy vegetables and fruit, was associated with higher bone density and higher bone mineral content in children [215].

In summary, many studies showed a positive association between the quantity of vegetable and/or fruit consumption and markers of bone health, or such an association was derived from the results of these studies. When solely those studies on direct evidence and studies with higher levels of evidence are taken into account, that is only prospective studies with the endpoints “osteoporosis” or “osteoporotic fracture”, there are currently only few studies available. Furthermore, these studies show inconsistent results. Therefore, the evidence regarding the *prevention of osteoporosis* due to a higher consumption of vegetables and fruit is judged as *possible*.

A similar judgement was reached by British experts based on a comprehensive literature review. It was concluded that a protective effect of a high intake of vegetables and fruit on bone health is to be regarded as possible, but the cause of this effect could not be determined [216].

### Eye diseases

Based on WHO data [217], it is assumed that more than 28 million subjects in Europe are visually impaired, with a prevalence for blindness of 0.3 %. The main causes for loss of sight in Europe and the United States are age-related macular degeneration (AMD; 50 %), glaucoma (18 %), diabetic retinopathy (17 %), and cataract (5 %) [218]. Despite worldwide trends for reduced prevalence of visual impairment and blindness since the 1990s [219], the prevalence of eye diseases in the ageing population is expected to increase in Western countries, for example, Germany, within the next 20 years [220]. The prevalence is reported to be 3.5–40 % depending on age for AMD, 3.3–14 % for glaucoma, and 4.4–20.9 % for diabetic retinopathy [221]. The prevalence for cataract increases with age and is over 40 % in subjects older than 75 years [222–224].

*Macular degeneration* is an age-related degenerative retinal disease that leads to the loss of central vision [225]. Risk factors for the development of AMD include age, smoking, and nutrition [226–228]. Important protective factors are dietary fibre [229], mono-unsaturated fatty acids [230], certain vitamins [231–233], and especially carotenoids like lutein and zeaxanthin. These carotenoids selectively accumulate in the *macula lutea* (point of high-resolution vision) and protect the pigment epithelial cells from blue light and damage by short-wave rays [234].

The dietary intake of carotenoids, the serum levels, and the supplementation of these carotenoids are associated with a risk reduction for AMD in most of the studies [235–244]. While protective effects of a high lutein/zeaxanthin intake were observed, one study showed an increased risk of AMD at high β-carotene intake [244].

In an analysis of the NHS [245, 246] and in the prospective *Rotterdam Study* [247], the intake of lutein/zeaxanthin and other carotenoids was not associated with the risk of AMD. The results of the *CHARM Study* (*Cardiovascular Health and Age*-*Related Maculopathy*) indicate that at already existing AMD, high lutein/zeaxanthin intake can promote AMD progression [248].

Although lutein/zeaxanthin intake was calculated directly from food intake in the mentioned studies, hardly any studies have been published that have investigated the association between vegetable and fruit consumption and risk of AMD. In a prospective cohort study, the consumption of fruit, but not of vegetables, was associated with a risk reduction by 36 % [249]. In women younger than 75 years, the risk of AMD was reduced by 52 % at higher intake of vegetables (4 vs. 0.9 portions per day) [250]. High intake (>5 times/week) of foods rich in lutein, like spinach and collard greens, was associated with a reduction in the AMD risk by 86 % in a case–control study [251]. According to Goldberg et al. [252], the intake (>7 times/week) of vegetables and fruit rich in provitamin A was associated with a reduction in the AMD risk by 33 % in a cross-sectional study.

*Cataract* is a clouding of the lens in adults, which results in impaired vision or visual acuity [253]. The risk is influenced by age, ethnic origin, gender, smoking, sunlight, consumption of alcohol, diabetes mellitus, corticosteroid medication, and nutritional factors [254]. The data on the influence of vitamin C and carotenoids on risk of cataract are inconsistent [255–259]. The more recent results of the prospective *Blue Mountains Eye Study* [260] suggest that high intake of vitamin C, especially from fruit juices, is associated with a significantly reduced risk of cataract. The combined intake of vitamin C and other antioxidants (β-carotene, vitamin E, zinc) from foods and/or supplements was also associated with a reduction in the cataract risk by 38–49 %.

In 4 prospective cohort studies, the influence of vegetable and fruit consumption on risk of cataract was investigated. A diet according to the *Dietary Guidelines for Americans* *2000* is associated with a cataract risk reduction by more than 50 % [261]. In this subpopulation of the *NHS*, eating habits and cataract were investigated in 479 women between the age of 52 and 73 years. In the group with the highest fruit consumption (3.9 portions/day), the prevalence of cataract was 42 % lower than in the control group (1.3 portions/day). In the HPFS, high consumption of broccoli and spinach in men was associated with a reduction in the cataract risk by 23 and 27 % [262]. In participants of the *Women’s Health Study,* a high intake of vegetables and fruit was associated with a significant reduction in the cataract risk by 10–15 % [263]. In the updated analysis of the same study [264], the risk reduction (10 %) at high intake of vegetables and fruit was not significant any more (changed database and analysis). In contrast, in the highest quintile of both lutein/zeaxanthin and vitamin E intake, the risk of cataract was 18 % and 14 % lower than in the lowest quintile of intake. In the *Carotenoids in the Age*-*Related Eye Disease Study* (*CAREDS*), the risk was reduced by 26 % at high vegetable intake [265]. Comparing highest with lowest quintiles, the risk of cataract was reduced by 32 % regarding both the calculated daily intake of lutein and zeaxanthin and the measured plasma concentrations of lutein and zeaxanthin. These findings are confirmed by results of the prospective *POLA Study* (*Pathologies Oculaires Liées à l’Age*) [242]. In the group with the highest plasma concentration of zeaxanthin (≥0.09 μM), the risk of cataract was reduced by 43 % compared with the control group (<0.04 μM).

*Glaucoma* is caused by changes in the intraocular pressure that can damage the optic nerve and may progress to complete blindness [266]. There are hardly any data on the influence of lifestyle factors on the risk of glaucoma. Regarding nutritional factors, so far mainly the role of vitamins has been investigated [267, 268]. Only one study described the association between vegetable and fruit intake and the risk of glaucoma [269]. In this cross-sectional investigation, a lowered risk was observed at high intake of certain kinds of vegetables and fruit, for example fresh carrots (−64 %).

*Diabetic retinopathy* is a microvascular complication of diabetes mellitus that is characterised by damage of the retina. Since it is a secondary disease of diabetes mellitus, there is a direct causal relation with the underlying primary disease [270, 271]. Currently, there are no studies available regarding the influence of the consumption of vegetables and fruit on the risk of diabetic retinopathy. Only in a small cross-sectional study from the *Melbourne Collaborative Cohort Study*, the association between the plasma concentration of carotenoids and the prevalence of diabetic retinopathy was investigated in 111 participants [272]. Type 2 diabetes patients with a diagnosis of diabetic retinopathy showed lower plasma concentrations of non-provitamin A carotenoids (lutein, zeaxanthin and lycopene) than patients without retinopathy.

Due to the low number of published studies, the evidence regarding the prevention of *macular degeneration* and *cataract* through higher consumption of vegetables and fruit is judged as *possible*. The evidence regarding the risk of *glaucoma* and *diabetic retinopathy* is *insufficient* due to the lack of data.

### Dementia

Dementia is a clinical syndrome that is characterised by a decrease in intelligence, memory, and perception and may be caused by various diseases. Logical and critical thinking, judgement, retentive memory, and short-term memory are impaired, while remote memory (long-term memory) can remain for a long time. In addition, personality may deteriorate [273].

According to the latest Report of the European College of Neuropsychopharmacology and the European Brain Council, 6.34 million people in Europe aged at least 60 years were estimated to suffer from dementia in 2011, corresponding to a mean prevalence of 5.4 % in this population. The prevalence is age-dependent (1–30 %) and increases with advanced age [274]. Due to increasing life expectancy in industrialised countries and the exponential increase in dementia in old age, the prevalence of dementia in these countries will be rising steadily. At global level, with an incidence of 4.6 million per year, an increasing prevalence of dementia with 42 million cases in 2020 has also to be expected [275]. Thus, dementia has become one of the major challenges to public health [276].

Worldwide, Alzheimer’s disease and vascular dementia are the two most common subtypes of dementia, which account for 50–70 and 15–25 % of all dementia cases, respectively [276]. Old age and genetic susceptibility are well established risk factors for dementia and Alzheimers’s disease. Vascular risk factors (e.g. diabetes mellitus, hypertension, and smoking) as well as cardio- and cerebrovascular diseases may contribute to the development and progression of dementia, whereas social, physical, and mental activities may delay their onset [276]. Overweight increases the risk of dementia independent of comorbidities [277].

So far, only a few studies have investigated whether the consumption of vegetables and fruit is associated with the risk of dementia. In addition to dementia, the cognitive performance has also been used as target parameter by using certain tests that are sensitive enough to diagnose dementia (both vascular and Alzheimer‘s dementia) at an early stage [278].

Cross-sectional studies in Spain [279] and Korea [280] have shown that elderly people with good cognitive performance consumed more fruit and vegetables than elderly people with impaired or poor cognitive performance. For the Korean participants, these differences regarding vegetable and fruit consumption were only detected in women; in men, they were only found regarding fruit intake, but not regarding vegetable consumption [280]. Vegetable consumption, however, was comparable in both study populations (Korea: 248 g/day vs. Spain: 239 g/day). In an Indonesian cross-sectional study, the consumption of fruit (including fruit juices), but not of orange/red and green vegetables, was significantly associated with improved short- and long-term memory in elderly people [281].

A cohort study including 3,718 participants of the *Chicago Health and Aging Project* (mean age at baseline 74 years) investigated the relation between the consumption of vegetables and fruit and the decrease in the cognitive performance (6 years follow-up) [282]. Cognitive performance was quantified using various screening methods. If vegetable and fruit intake was analysed in total, there was not any association between the number of consumed portions and cognitive deficits, while an inverse association was found for the consumption of vegetables alone, but not for the consumption of fruit. Two further cohort studies showed similar results. The cohort study by Kang et al. [283] investigated the decline in cognitive performance in a subgroup of the NHS (age at baseline: 30–55 years, follow-up: 19–25 years). Vegetable and fruit consumption including fruit juices were recorded. The decline in cognitive performance was inversely associated with vegetable consumption, but not with the intake of fruit or fruit juices. In a Dutch cohort (*n* = 2,613, age at baseline: 43–70 years, follow-up: 10 years), an inverse association was also found only between vegetable consumption and cognitive performance, but not regarding fruit and juices [284]. In contrast to the study by Nooyens et al. [284], the studies by Morris et al. and Kang et al. [282, 283] showed an association between the consumption of green leafy vegetables and a reduced risk, but not for other types of vegetables (yellow vegetables and cruciferous vegetables). Nooyens et al. [284] detected an inverse association between the consumption of root vegetables (carrots, beetroot) and the decrease in cognitive performance. While the results of cross-sectional studies show a protective effect of both fruit and vegetables for maintaining the cognitive performance [279–281], the results of cohort studies suggest only a protective effect regarding vegetables.

To date, 3 cohort studies with dementia or Alzheimer’s dementia as target parameter have been performed [285–287]; a fourth cohort study [288] also included cognitive impairment in addition to dementia. Dai et al. [285] determined the intake of vegetable and fruit juices in 1,836 Japanese immigrants (mean age, 71 years) between 1992 and 1994 in relation to the incidence of Alzheimer’ dementia in 2001. The risk of disease decreased with increasing consumption, independent of the intake of vitamin C, E, and β-carotene [285]. Barberger-Gateau et al. [286] investigated the frequency of vegetables and fruit consumption in 8,085 subjects (aged ≥65 years) in Bordeaux, Dijon, and Montpellier (France). After 3.6 years follow-up, the frequency of vegetable and fruit consumption reduced the risk of dementia, including the risk of Alzheimer’s dementia. Daily consumption compared with rare consumption was associated with a risk reduction by about 30 %. Similar results were found in the study of Hughes et al. [287], which investigated only 3,779 individuals within the *Swedish Twins* (*HARMONY*) *Study* (mean age at baseline: 48 years), but had a follow-up of 30 years. In this study, the medium or high intake of fruit and vegetables was associated with a decreased risk of dementia and Alzheimer’s dementia compared with low or no consumption of fruit and vegetables. However, this difference was only significant in fully adjusted models. Compared with the consumption of more than 2 portions of vegetables and fruit per day, the consumption of <2 portions was associated with a significantly higher risk of dementia including cognitive impairment in the cohort of the *Esprit Study* (age at baseline: ≥65 years, median follow-up: 7 years) [288], taking into account age and gender.

In summary, the studies on cognitive performance and risk of dementia suggest an inverse relation to the consumption of vegetables and fruit. Due to the limited number of studies, *possible* evidence exists for a reduced risk of dementia with increasing consumption of vegetables and fruit. In this respect, the consumption of vegetables seems to be more important than the consumption of fruit.

## Summary of the evidence judgement

The current evaluation of the association between the consumption of vegetables and fruit and the risk of certain diseases resulted in all grades of the strength of evidence.

For hypertension, CHD, and stroke, there is *convincing evidence* that increasing consumption of vegetables and fruit reduces the risk of disease. The setting of the strength of evidence for hypertension as convincing is particularly important, because hypertension is widespread and is considered as a risk factor of CHD and stroke. There is *probable evidence* that the overall risk of cancer is inversely associated with the consumption of vegetables and fruit. The evidence was not individually judged for the different sites of cancer, as this would go beyond the scope of this review due to the various aetiologies. Data on dementia indicate *possible evidence* for a risk-reducing influence of increased vegetable and fruit consumption. In addition, there is *possible evidence* that a diet with increased consumption of vegetables and fruit may prevent body weight gain. As overweight is the most important risk factor for type 2 diabetes mellitus, an increased consumption of vegetables and fruit might indirectly reduce the incidence of type 2 diabetes mellitus. However, there is *probable evidence* that there is *no influence* of increased consumption of vegetables and fruit on the risk of type 2 diabetes mellitus that is independent of overweight.

Furthermore, the present data indicate that an increased consumption of vegetables and fruit also reduces the risk of certain eye diseases, RA, and osteoporosis. Likewise, the present data indicate that an increase in vegetable and fruit consumption may contribute to the prevention of the lung diseases asthma and COPD. Because of the chosen evidence criteria and the lack of studies with level of evidence I and II, the evidence regarding the association between increased vegetable and fruit consumption and reduced risk of these diseases is only judged as *possible*. For chronic IBDs, glaucoma, and diabetic retinopathy, evidence is *insufficient*. Table 3 summarises the evidence judgement regarding the influence of vegetable and fruit consumption on the risk of certain chronic diseases.

**Table 3:** Summary of the strength of evidence on the association between the consumption of vegetables and fruit and the risk of chronic diseases

	Evidence judgement (strength of the evidence)			
	Convincing	Probable	Possible	Insufficient
Obesity		o^a^	↓^b^	
Type 2 diabetes mellitus		o		
Hypertension	↓			
Coronary heart disease (CHD)	↓			
Stroke	↓			
Cancer		↓		
Chronic inflammatory bowel diseases				~
Rheumatoid arthritis (RA)			↓	
Chronic obstructive pulmonary disease (COPD)			↓	
Asthma			↓	
Osteoporosis			↓	
Eye diseases				
Macular degeneration			↓	
Cataract			↓	
Glaucoma				~
Diabetic retinopathy				~
Dementia			↓	

↓ Risk reduction by increased vegetable and fruit consumption, o no association, ~ insufficient evidence

^a^Weight loss

^b^Weight increase

## Discussion

The present review shows a considerable preventive potential of an increase in consumption of vegetables and fruit by the general public in respect to a number of diseases. The chain of evidence according to the criteria of a risk-reducing effect with convincing evidence is well reflected in the available data on hypertension, CHD, and stroke. In contrast to these diseases, the risk-reducing effect of consumption of vegetables and fruit for cancer is assessed to be much smaller than in earlier evaluations (e.g. [94, 97]) taking the present review and the current literature [102, 289]. It should be noted that the strength of evidence neither indicates the degree of risk reduction nor the intake quantity that is necessary to achieve the risk-reducing effect. Such information cannot be derived from the presented data and is not subject of this review.

The scientific basis of the “5 a day” campaign, which has been greatly promoted in recent years in Europe and nationwide, is strongly supported by the prevention potential demonstrated here. There seems to be a broader basis for disease prevention than assumed at the beginning of the campaign. At the establishment of the Private Public Partnership at the beginning of the 1990s between science—with the National Cancer Institute of the USA as leading institution—and the food industry, the focus was primarily on cancer. Nowadays, the recommendation to increase the consumption of vegetables and fruit is mainly based upon the convincing data regarding hypertension, CHD, and stroke and the potential for many other diseases.

However, not only the “5 a day” campaign profited from the progress in the available data, but also the scientific societies focusing on other diseases besides cancer. For example, the European Society of Cardiology promotes the intake of vegetables and fruit in its guidelines about prevention [290].

The changed data situation on the association between the intake of vegetables and fruit and the risk of cancer is of great scientific interest. In the systematic review by Block et al. [93], case–control studies were predominant, and 128 of the 156 studies available at that time showed an inverse risk relation. Unlike case–control studies, prospective cohort studies do not record the consumption of vegetables and fruit retrospectively after the disease has occurred, but at the time of study entry without already existing disease; studies like these were predominantly published after the year 2000 and showed inconsistent results. They also showed a much lower risk association than the case–control studies [97]. This led to the assumption that there was a considerable systematic bias regarding the retrospective documentation of food intake in the case–control studies. But perhaps, the methodological problems of the study design are not the only possible explanation for the change in the data situation in the recent decade. The change in the data situation might also be due to alterations in lifestyle in recent decades, which, on the one hand, resulted in an improved supply with essential nutrients and, on the other hand, led to a decrease in carcinogenic or tumour growth-promoting factors. It has to be considered that the difference in age in the case–control studies compared with the cohort studies may be much larger than the 10 years mentioned above in which the change in the strength of evidence occurred, as most of the participants in case–control studies are older diseased subjects, while there are mainly younger healthy individuals in cohort studies. The current analysis of the *EPIC Study* on the impact of consumption of vegetables and fruit on the prevention of cancer indicates that the largest reduction in risk was observed in cancer sites that are promoted by smoking [106]. Obviously, a lifestyle that is associated with higher exposure to carcinogens may allow a stronger cancer preventive effect of vegetables and fruit than a lifestyle with lower exposure to carcinogens. This might be an additional explanation for the observed small risk reduction in the more recent prospective studies.

Changes in lifestyle have resulted in a massive increase in the prevalence of obesity in Western countries. Obesity-related cancer-promoting mechanisms include chronic inflammation, insulin resistance, impaired glucose tolerance, and altered hormone metabolism [291, 292]. Vegetable and fruit intake has proven to influence these processes. For example, an increased consumption of vegetables and fruit can counteract chronic subclinical inflammatory processes involved in cancer and obesity [293]. Greater botanical variety in vegetable and fruit intake is associated with less inflammation [294].

It has been shown that the consumption of vegetables and fruit from certain botanical families exerts special protective effects against various cancers (like lung cancer), which do not become obvious if all kinds of vegetable and fruit are analysed together [295]. Similar observations were made for specific subtypes of cancer. Vegetable and fruit consumption showed a protective effect only against certain types of lung cancer (squamous cell carcinoma), but not against other histological types of lung cancer [296, 297]. Therefore, the overall analysis of all vegetable and fruit kinds and all cancers can result in a serious loss of information.

As vegetables and fruit and the phytochemicals therein particularly influence not only inflammatory processes, but also cellular redox processes as well as endothelial and metabolic processes [83–88], which are involved in the pathogenesis of various diseases, we assume that these mechanisms are primarily responsible for the risk-reducing effect of vegetable and fruit consumption regarding the single diseases. This also applies to diseases with limited data so far, for which the strength of evidence grade “possible” was assigned.

Therefore, it seems to be reasonable to perform intervention studies that specifically contribute to the elucidation of these mechanisms. As it is often not possible to implement well-controlled long-term dietary changes in a randomised study design, at first, intervention studies should be designed as short-term studies with the investigation into appropriate surrogate markers of hard endpoints. These intervention studies should use the full range of available vegetables and fruit as much as possible. Furthermore, it is necessary to systematically continue the data analyses in the present cohort studies regarding the associations between vegetable and fruit consumption and the risk of various diseases. There also seems to be the need to critically review the data on the assessment of the consumption of vegetables and fruit and to improve the methods if possible [298]. The results from prospective cohort studies together with those from intervention studies on the mechanisms of action will provide a sound basis for future evaluations of the preventive potential of vegetable and fruit consumption regarding various chronic diseases using an evidence-based approach.

## Footnotes

1 Overweight: BMI ≥ 25.0; pre-obesity: BMI 25–29.9; obesity: BMI ≥ 30.0 (according to [10]).

2 Overweight: >90th percentile of the BMI (according to [11]).

3 Obesity: >97th percentile of the BMI (according to [11]).

4 Normal weight: BMI between the 10th and 90th BMI-percentile (according to [11]).

5 The DASH (*Dietary Approaches to Stop Hypertension*) diet consists of a high proportion of vegetables and fruit, low-fat dairy products and cereal products high in dietary fibre, combined with a low-fat proportion (<30 energy %).
